# Leukocyte oxygen radical production determines disease severity in the recurrent Guillain-Barré syndrome

**DOI:** 10.1186/1476-9255-7-40

**Published:** 2010-08-08

**Authors:** Natalia Mossberg, Oluf Andersen, Magnus Nordin, Staffan Nilsson, Åke Svedhem, Tomas Bergström, Kristoffer Hellstrand, Charlotta Movitz

**Affiliations:** 1 Department of Neuroscience and Physiology, University of Gothenburg, Sweden; 2 Department of Infectious Diseases, University of Gothenburg, Sweden; 3 Department of Mathematical Statistics, Chalmers University of Technology, Gothenburg, Sweden

## Abstract

**Background:**

The recurrent Guillain-Barré syndrome (RGBS) is characterized by at least two GBS episodes with intervening remission. In a previous study of monophasic GBS, we reported that the magnitude of oxygen radical production ("respiratory burst") in peripheral blood leukocytes was inversely correlated to disease severity. The present study sought to establish a similar correlation in patients with RGBS.

**Methods:**

Oxygen radical production in leukocytes was induced by formyl-Met-Leu-Phe (fMLF), Trp-Lys-Tyr-Met-Val-Met-NH_2 _(WKYMVM), or phorbol myristate acetate (PMA) and assessed by quantifying superoxide anion formed by the leukocyte NADPH oxidase.

**Results:**

Disease severity, assessed using the MRC score, was negatively correlated to superoxide anion production triggered by fMLF or WKYMVM (p = 0.001 and 0.002, respectively; n = 10). Superoxide anion production also was significantly lower in RGBS patients with incomplete recovery after stimulation with fMLF (p = 0.004) or WKYMVM (p = 0.003).

**Conclusion:**

We conclude that a lower respiratory burst in leukocytes is strongly associated with a severe course of RGBS.

## Introduction

The recurrent Guillain-Barré syndrome (RGBS) is a demyelinating disease of the peripheral nervous system characterized by episodes of relapsing and remitting symptoms with complete or near complete functional recovery between episodes [1-3]. Each episode fulfils the diagnostic criteria for GBS including symmetric limb weakness and decreased or absent muscle reflexes, or Miller Fisher syndrome [4,5].

The respiratory burst in human phagocytes is a pivotal anti-microbial effector function of the innate immune defense [6]. The nicotinamide adenine dinucleotide phosphate (NADPH) oxidase initiates the respiratory burst by reducing oxygen in phagocytic cells to form reactive oxygen species such as superoxide anions, hydrogen peroxide, toxic halids, and hydroxyl radicals. While it is well established that these toxic oxygen derivatives participate in controlling bacterial and parasitic infections, a deficiency in NADPH oxidase activity may also be related to the development of autoimmunity. A polymorphism in *Ncf1*, which encodes a cytosolic oxidase component, compromises NADPH oxidase function and predisposes for a severe course of experimental autoimmune encephalitis, arthritis, and neuritis in rodent models [7-9].

In patients with the monophasic form of GBS, a low capacity of blood leukocytes to generate oxygen radicals in response to stimulation with NADPH oxidase-activating agents was recently found to correlate with a severe course of disease [10]. A similar correlation has also been found in multiple sclerosis [11]. The present study was designed to determine a putative relationship between the intensity of respiratory burst in leukocytes and disease severity in patients with RGBS.

## Materials and methods

Trp-Lys-Tyr-Met-Val-Met-NH_2 _(WKYMVM) was from Alta Bioscience (University of Birmingham, United Kingdom). N-formyl-Met-Leu-Phe (fMLF) and phorbol myristate acetate (PMA) were from Sigma Chemical Co. (St. Louis, Missouri). Horseradish peroxidase (HRP) was from Boehringer-Mannheim (Mannheim, Germany). Luminol and isoluminol were from Sigma Chemical Co.

### Study subjects and diagnostic criteria

RGBS patients were included according to the following definition: at least two episodes of relapsing symmetric limb weakness with decreased or absent muscle reflexes, where each episode fulfilled the diagnostic criteria for GBS with time from onset to peak neurological deficit (time to nadir) of four weeks or less [4,5]. The recovery after each relapse should be complete or near complete [3], with a minimum of at least two months between relapses [12]. Patients with treatment related fluctuations [13] and patients with the recurrent form of chronic inflammatory demyelinating polyneuropathy (CIDP), in whom time to nadir is more than 2 months, were excluded [14].

Ten consecutive RGBS patients (nine males and one woman, aged 43) in remission and without immunomodulatory therapy, admitted to Sahlgrenska University Hospital during relapses, were included for follow-up examination. All patients had been treated during attacks with intravenous immunoglobuline or plasma exchange, and two patients had previously received prophylactic immunosuppressive therapy. Healthy controls were the patients' spouses (n = 5) or age-matched subjects (n = 5; mean age 52, range 33-69 years). The study was approved by the Medical Ethics Committee and written informed consent was obtained from all participants.

### Clinical examination and retrospective evaluation

A neurological follow-up examination was performed with scoring according to Medical Research Council sum score (MRC) [15], and the degree of remission (complete or incomplete) was recorded for the second and last attack. MRC score of 60 (maximum in healthy persons) is defined as a complete recovery. At follow-up the patients were free from relapse. Nine patients had not received immunomodulatory treatment for at least one month before examination and blood sampling, and one patient (with a history of ulcerative colitis) had received low dose cortisone (10 mg/day) until 1 week prior to blood sampling. The median time from the last relapse to examination and sampling was 7 months (range 1 month-12 years). Information of antecedent events, number of episodes and interval between episodes, time to second episode, age at first and last episode, duration of the plateau phase, episode duration, and the degree of remission for each episode were obtained from medical records. Neurophysiological follow-up examination including nerve conduction study was performed according to routine methods used at the Department of Clinical Neurophysiology at Sahlgrenska University Hospital.

### Leukocyte isolation

Peripheral leukocytes (neutrophils, monocytes, and lymphocytes) were isolated from heparinized venous blood by dextran sedimentation and hypotonic lysis of remaining erythrocytes as described elsewhere [16,17]. Leukocytes were resuspended (10^7 ^cells/ml) in Krebs-Ringer buffer (KRG) [18]. No significant differences in leukocyte counts or distribution were detected between patients and controls or between groups of patients with different severity of RGBS (data not shown). Sorting experiments revealed that > 99% of the oxygen radical production in peripheral blood leukocytes was contributed by phagocytic cells, i.e. monocytes and neutrophilic granulocytes (data not shown).

### NADPH oxidase activity

Leukocyte production of superoxide anion, which is the initial oxygen radical formed by the NADPH oxidase [18], was assessed in duplicate using isoluminol/luminol-enhanced chemiluminescence (CL) technique [19]. The CL was measured in a Biolumat LB 9505 (Berthold Co., Wildbad, Germany), using a 1 ml reaction mixture containing 10^6 ^leukocytes, horseradish peroxidase (4 U/ml), and isoluminol or luminol (20 μM). The cells were activated by 10^-7 ^M fMLF, 10^-7 ^M WKYMVM or 5 × 10^-8 ^M PMA.

### Myeloperoxidase activity

Myeloperoxidase (MPO), a leukocyte enzyme, forms hypochlorous acid from H_2_O_2 _which results in the production of toxic halides and the hydroxyl radical [20]. MPO activity was assessed in leukocytes (KRG, 10^7 ^cells/ml) and measured spectrophotometrically as the oxidation of 4-aminoantipyrine in the presence of hydrogen peroxide and MPO [17]. Volumes of 430 μl 2.5 mM 4-aminoantipyrine and 500 μl 1.7 mM H_2_O_2 _were added to cuvettes. After equilibration for 4 min at room temperature, 100 μl sonicated cells (2 × 10^6 ^cells/ml), pre-incubated with 0.1% Triton X-100, were added. The change in absorbance was measured at 510 nm for 4 min (Perkin Elmer lambda 2) in duplicate and the mean value was used for statistical analysis.

### Microbiological analyses

A standardized set of serological analyses for diagnosis of viral and bacterial infections were performed at the Department of Virology, Sahlgrenska University Hospital. Serum IgG and IgM analyses for cytomegalovirus (CMV), Epstein-Barr virus (EBV), herpes simplex virus (HSV), varicella zoster virus (VZV), mumps, measles, rubella, enterovirus, influenza A and B, parainfluenza, respiratory syncytial virus (RSV), adenovirus and *Mycoplasma pneumoniae *were performed in the acute and convalescent phases of several RGBS attacks. The following methods were used: enzyme-linked immunosorbent assay (ELISA) for IgG and immunofluorescence (IF) assay of IgM antibodies against HSV (type 1 and 2), VZV and CMV; by IF assay of IgG and IgM antibodies against EBV, mumps, measles, and *Toxoplasma gondii*; by ELISA of IgM for enteroviruses, rubella, RSV and *Mycoplasma pneumoniae*; and by complement fixation (CF) assay of antibodies against influenza A and B. Criteria for positive diagnosis of these infections were either at least a four-fold titre-rise between paired samples (IgG and CF) or positive IgM at more than four-fold dilutions over negative controls. The assay of *Campylobacter jejuni *infection was based on a DIG-ELISA utilising an outer membrane glycoprotein. Criteria for diagnosis were positive IgM or IgA titres or a significant rise of consecutive IgG titres [21].

### Statistics

Superoxide anion production and MPO activity were analyzed as response variables with a linear model using RGBS severity groups as predictors with a one-sided research hypothesis based on previous findings for GBS. The measurement day was used as a nuisance factor to adjust for experimental day-to-day variation. A paired test was used between patients and controls. For numeric severity variables the relationship to superoxide anion production was analyzed by one-sided Pearson (r) or Spearman (r_s_) correlation. The logarithm of the time to the second bout was used in correlation analysis.

## Results

### Clinical data

The median time between the first attack and follow-up was 15 years (0.5-40 years). A total of 38 attacks occurred in these patients (median 3 per patient, range 2-7) (Table 1). Five patients had complete recovery and 5 patients had motor residual deficit at follow-up.

**Table 1:** RGBS patient characteristics

Clinical data	RGBS patients, n = 10
Mean number episodes per patient	3 (2-7)

Follow-up time since first episode	15 years (0.5-40 years)

Mean age at follow-up	52 years (33-73 years)

Mean age first episode	35.7 years (21-52 years)

Respiratory care	3 patients, 7 episodes

**Preceding infection** **at relapse**	

URTI	8 patients, 22 episodes

*Campylobacter jejuni*	5 patients, 13 of 22 episodes*

CMV	3 patients, 4 of 5 episodes*

RSV	1 patient, 3 of 4 episodes*

Influenza A	1 patient, 1 of 3 episodes*

VZV reactivation	1 patient, 1 of 4 episodes*

URTI- upper respiratory tract infection

* Number of episodes with a positive virus- or *Campylobacter*

serology per number of episodes with adequate (according to section

*Microbiological analyses*) sampling and analysis.

Only positive findings were included in this table.

### Preceding infections in relation to relapse

Several relapses were preceded by an infectious illness, most commonly by an upper respiratory tract infection (present in 8 patients) or a *Campylobacter *infection (present in 5 patients and detected in 13 of 22 episodes) (Table 1).

### Correlation between clinical aspects of RGBS and respiratory burst

**Figure 1:**
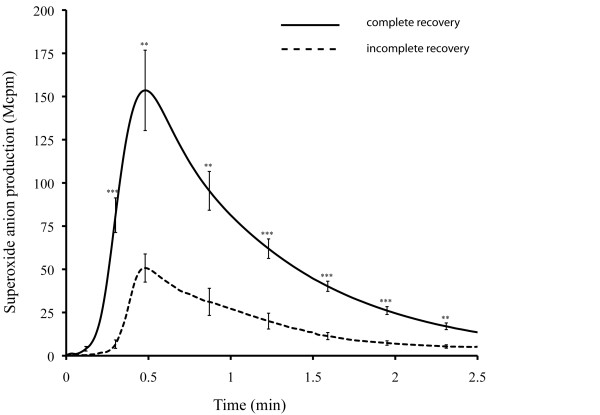
**Production of superoxide anion in leukocytes isolated from RGBS patients in relation to complete and incomplete remission**. Leukocytes isolated from RGBS patients were stimulated by 10^-7 ^M fMLF. The production of superoxide anion was measured kinetically by isoluminol or luminol-amplified chemiluminescence. Responses are given as Mcpm (10^6 ^counts per minute), presented as the mean CL values ± SEM at different time points. The statistical significance of difference in superoxide anion production between subjects with complete (solid line) and incomplete recovery (dashed line) was determined using two-tailed, unpaired Student's t-test, p < 0.01**, p < 0.001***.

The superoxide anion production after stimulation with fMLF and WKYMVM was significantly lower in RGBS patients with incomplete than complete recovery after the second attack (p = 0.0035 for both peptides) and at follow-up (p = 0.004 and p = 0.003; Table 2 and Fig. 1). Superoxide anion production was correlated with MRC score at follow-up when induced by fMLF (r_s _= 0.85, p = 0.001) and by WKYMVM (r_s _= 0.82, p = 0.002). Lower superoxide anion production after stimulation with fMLF and WKYMVM was associated with a longer plateau phase during relapse (r = -0.59, p = 0.037 and r = -0.58, p = 0.04), a shorter median interval between relapses (r = 0.68, p = 0.016 and r = 0.76, p = 0.006), and a shorter interval to the second attack (r = 0.58, p = 0.039 and r = 0.66, p = 0.02). There was no significant difference in superoxide anion production between RGBS patients and their age-matched controls, for whom the superoxide anion production was intermediate between patients with complete and incomplete recovery (p = 0.09 for fMLF, p = 0.19 for WKYMVM and p = 0.23 for PMA).

**Table 2:** Superoxide anion production in leukocytes isolated from RGBS patients with incomplete and complete recovery at follow- up

	*O_2_^- ^production (Mcpm)*		
**stimuli**	**- recovery**	**+ recovery**	**p value**

fMLF	47 ± 23	170 ± 25	0.004

WKYMVM	54 ± 20	166 ± 21	0.003

PMA	488 ± 35	576 ± 38	0.08

Leukocytes isolated from RGBS subjects were triggered with fMLF (10^-7 ^M), WKYMVM (10^-7 ^M) or PMA (5 × 10^-8 ^M). The release of superoxide anions was measured by isoluminol/luminol-enhanced CL. Data are presented as marginal mean peak values ± SEM comparing O_2_^-^ production between patients with incomplete (-) and complete (+) recovery using a one-sided test in a linear model.

A lower superoxide anion production after stimulation with fMLF and WKYMVM was associated with a longer distal motor latency (r_s _= -0.74, p = 0.007 and r_s _= -0.69 and p = 0.015), a lower motor nerve conduction velocity (r_s _= 0.59, p = 0.037 for both peptides), and a lower amplitude (r_s _= 0.58, p = 0.037 and r_s _= 0.69, p = 0.013) at follow-up.

### MPO activity

MPO activity in leukocytes did not differ between patients and controls (p = 0.63) or between severity groups of RGBS patients with complete or incomplete recovery (p = 0.57) (data not shown).

## Discussion

A main finding in this study was that superoxide anion production by leukocytes in response to NADPH oxidase-activating peptides was associated with severity of disease in patients with RGBS. Thus, the superoxide anion response to fMLF and WKYMVM was significantly lower in RGBS patients with incomplete recovery, higher residual motor deficit, and longer duration of the plateau phase during attacks. We also observed a significant association between the individual attack frequency (shorter interval between relapses including the time from the onset to the second relapse) and respiratory burst, indicating a higher tendency for the disease to recur in patients with low superoxide anion production.

The peptides fMLF and WKYMVM activates NADPH oxidase via formyl peptide receptors (FPRs), which are transmembrane G-protein coupled receptors present in monocytes and neutrophils. fMLF interacts with FPR1, whereas WKYMVM binds to FPR2 and FPR3 [22]. Notably, the severity of RGBS was unrelated to the oxygen radical production in response to PMA, a membrane-permeable proteine kinase C (PKC) activator [23]. PKC is located downstream of FPR1, FPR2 and FPR3 and upstream of the NADPH oxidase, and thus activates the oxidase independently of FPRs. Since no difference in MPO activity was detected, the difference in radical production is not dependent on the H_2_O_2 _(produced by the NADPH oxidase) consuming MPO-H_2_O_2_-halide system. Our findings therefore suggest that the overall function of the oxidase is intact in patients with severe RGBS, and that the reduced responsiveness to NADPH oxidase inducers in patients with severe RGBS is located upstream of PKC.

These findings imply that the capacity of leukocytes to mount a respiratory burst is of relevance to the severity of autoimmunity, as previously shown in monophasic GBS and in MS [10,11]. In this regard GBS, RGBS and MS mirror previous studies in rodents, in which a deficient NADPH oxidase was associated with severity of experimental autoimmunity [7-9]. The mechanisms underlying the association between low oxygen radical production and severe symptoms of autoimmunity disease are not known. In accordance with previous studies [1], we found a high incidence of infections prior to onset of RGBS, and it may be speculated that a reduced oxygen radical production results in a less efficacious microbial clearance. An alternative or supplementary mechanism relates to the regulatory effects of oxygen radicals on T cells and other subsets of lymphocytes. Phagocyte-derived oxygen radicals efficiently trigger apoptosis in CD4^+ ^and CD8^+ ^T cells [24,25], and it is conceivable that a deficient elimination of autoreactive T cells by oxygen radicals may aggravate autoimmune manifestations [26].

Oxygen radicals are traditionally believed to promote damage of tissue in several diseases including atherosclerosis, reperfusion injury, and emphysema [6]. ROS produced locally in CNS function as a mediator of demyelination and axonal injury, both in experimental autoimmune encephalomyelitis and MS [27,28]. However, there is increasing evidence for a protective role of innate immunity in autoimmune disease [29] in which oxygen radicals dampen the development of autoimmune disorders (reviewed in [30]). This is shown in Crohn's disease where a defect neutrophil function, including migration, chemotaxis, supeoxide anion production, and phagocytosis has been described [31]. The effects of a deficient oxygen radical production in chronic granulomatous disease (CGD) are interesting. CGD is caused by an inability of the NADPH oxidase to produce oxygen radicals and is characterized by recurrent infections, sterile granuloma but is also associated with autoimmune diseases [32,33]. In a recent study, CGD monocytes were shown to produce significantly higher levels of cytokines than control cells [34] which could explain the association with autoimmunity. It is thus important to discriminate between oxygen radicals and oxidative stress. The latest is a process of harmful massive production of oxygen radical with tissue destructive properties. On the other hand, oxygen radicals produced at the right time and place and with strict (redox) regulation may have a beneficial regulatory potential, playing a crucial role in the innate immune defence [35].

## Conclusion

In conclusion, we propose that the capacity of leukocytes to generate oxygen radicals in response to NADPH oxidase-triggering peptides is related to the severity of RGBS. The mechanistic aspects of this association should be the subject of further study, along with studies outlining the role of leukocyte-derived oxygen radical for other autoimmune diseases.

## Competing interests

The authors declare that they have no competing interests.

## Authors' contributions

NM, CM, OA, KH and TB participated in the conception and design of the study. NM and CM carried out the measurements of oxygen radical production. CM performed MPO activity measurements. NM and OA performed the clinical examination and retrospective evaluation. MN performed the neurophysiological follow-up examination. TB and ÅS participated in the microbiological analyses. SN and NM performed the statistical analysis. KH, CM, MN and OA drafted the manuscript. All authors read and approved the final manuscript.
